# The effect of glass shape on alcohol consumption in a naturalistic setting: a feasibility study

**DOI:** 10.1186/s40814-015-0022-2

**Published:** 2015-07-20

**Authors:** David M. Troy, Olivia M. Maynard, Matthew Hickman, Angela S. Attwood, Marcus R. Munafò

**Affiliations:** 1 MRC Integrative Epidemiology Unit (IEU), University of Bristol, Bristol, UK; 2 UK Centre for Tobacco and Alcohol Studies, School of Experimental Psychology, University of Bristol, 12a Priory Road, Bristol, BS8 1TU UK; 3 School of Social and Community Medicine, University of Bristol, Bristol, UK

**Keywords:** Alcohol, Glass shape, Harm reduction, Choice architecture

## Abstract

**Background:**

Alcohol-related harms are a major public health concern, and population-level interventions are needed to reduce excessive alcohol consumption. Glass shape is an easily modifiable target for public health intervention. Laboratory findings show beer is consumed slower from a straight glass compared to a curved glass, but these findings have not been replicated in a naturalistic setting. The purpose of this study is to investigate the feasibility of conducting a randomised controlled trial investigating the effect of glass shape on alcohol consumption in public houses.

**Methods:**

Straight and curved half-pint and pint glasses were delivered to three public houses over two weekends. Glass type was counterbalanced over the two weekends and between the public houses. Monetary takings were recorded as an indirect measure of consumption.

**Results:**

Replacing stocks of glassware in public houses was feasible and can be enacted in a short space of time. One landlord found the study too disruptive, possibly due to a laborious exchange of glassware and complaints about the new glassware from some customers. One public house’s dishwasher could not accommodate the supplied curved full-pint glasses. Obtaining monetary takings from public house staff was a feasible and efficient way of measuring consumption, although reporting absolute amounts may be commercially sensitive. Monetary takings were reduced by 24 % (95 % confidence interval 77 % reduction to 29 % increase) when straight glasses were used compared to curved glasses.

**Conclusions:**

This study shows that it is feasible to carry out a trial investigating glass shape in a naturalistic environment, although a number of challenges were encountered. Brewery owners and landlords are willing to engage with public health research in settings where alcohol is consumed, such as public houses. Good communication with stakeholders was vital to acquire good data, and highlighting the potential commercial benefits of participating was vital to the study’s success. A full scale evaluation of the effects of glass shape on alcohol consumption could inform local and national policy.

## Background

Excessive alcohol use, defined as consuming above the UK government’s recommended daily amounts of alcohol, is a major public health concern [1]. In England in 2012, among adults who had consumed alcohol in the last week, 55 % of men and 53 % of women drank more than the recommended daily amounts [2]. Liver disease is the fifth commonest cause of death and the only major cause in the UK that continues to rise [3]. The need to reduce excessive consumption of alcohol is pressing. Population-level interventions such as price increases [4–6] and reduced availability [7] are effective, but other approaches are required as part of a holistic approach to reducing alcohol consumption.

Choice architecture interventions have become popular among policy makers in recent years [8, 9]. By altering the environments within which people make choices, choice architecture interventions allow for behaviour to be influenced at the population level [10]. Choice architecture interventions alter the way choices are presented and/or the properties or placement of objects or stimuli within a micro-environment in an attempt to prompt healthier behaviours. Interventions of this type do not coerce, or prohibit any action, on the part of the individual. The advantages of these interventions are that they mainly rely on automatic psychological processes of the target individual [11–13] resulting in a positive impact regardless of individual differences. Choice architecture interventions which can be embedded within microenvironments where alcohol consumption occurs (e.g. public houses and bars) are likely to be particularly effective, given the extremely wide potential reach of such interventions. To date, however, choice architecture interventions have been directed at changing alcohol use in only a limited number of studies (7.3 % of studies in a recent review [10]). These studies focused on the effect of altering the ambience and functional design of drinking venues. Louder music was associated with higher alcohol consumption [14, 15]. Other characteristics of drinking venues, such as a permissive environment (i.e. ‘anything goes’ atmosphere, swearing, overt sexual contact, poor overall order at the premises), availability of cheap alcohol, poor cleanliness, crowding, a focus on dancing and poor staff practice were identified as contributing to alcohol-related violence, crime and harm (i.e. injuries, accidents), although these findings were not consistent across studies [16]. Glassware has been targeted as a potential area for intervention; a recent study showed that more alcohol was poured into short, wide glasses than tall, slender glasses by both students and bartenders [17]. Labelling alcohol products is another possible intervention, although evidence to date is mixed. Displaying accurate alcohol by volume (ABV) information, in isolation, has been suggested to *increase* heavy drinking in young people as they use it to assist them in choosing the strongest drinks for the lowest price [18]. While applying health warning labels to alcohol beverages in the US has resulted in greater awareness of alcohol-related harms, the impact on drinking behaviour has been minimal [19].

One possible choice architecture intervention is glass shape, which has been shown to alter the rate of alcohol consumption under laboratory conditions; beer consumed from a straight glass was consumed slower compared to a curved glass [20]. Intuitively, this slowing of drinking rate is likely to have two effects: reduced intoxication and reduced consumption overall. However, alcohol is normally consumed in a social context with many different and competing pressures influencing the drinker. People have been shown to imitate and be influenced by the drinking behaviour of others [21]. Personal expectations of alcohol influences consumption; alcohol expectancies (beliefs regarding the cognitive, affective and behavioural consequences of drinking [22]) have consistently been shown to affect alcohol use [23–26]. Positive outcome expectancies have been linked to problematic drinking [27–30], while negative outcome expectancies have been linked with lower alcohol consumption [31–33]. Increases in consumption have also been associated with negative outcome expectancies [34, 35] which suggests a more complex relationship is at work. The importance of testing in a naturalistic environment is that the expectancies of alcohol in a laboratory environment may be quite different from the expectancies in a bar, leading to differing alcohol use.

There are a number of potential challenges to carrying out a randomised controlled trial of glass shape on alcohol consumption in a naturalistic setting. These include the willingness of bars and public houses to participate in the trial, their compliance during the trial, the logistical challenges of changing glassware on a regular basis and the assessment of alcohol consumption via monetary takings. We therefore conducted a feasibility study to investigate these challenges.

## Methods

### Study design

The study was a feasibility study investigating the viability of manipulating the shape of glasses in a naturalistic setting and its effect on alcohol consumption. The study took place in three public houses over two weekends (Friday to Saturday nights, inclusive). Determining the feasibility of the study was the primary outcome and measuring monetary takings was the secondary outcome which provided an indirect measure of alcohol consumption. The exchange of glassware with the public houses was made the week before the first weekend and midweek between weekends. The types of glasses that we intended the public houses to use were counterbalanced over the two weekends and between the public houses, although the actual allocation differed somewhat (Table 1).

**Table 1:** Planned and actual glass conditions in the three public houses over both study weekends

	Weekend 1		Weekend 2	
	Planned	Actual	Planned	Actual
Pub 1	Curved	Normal glass range^a^	Straight	Straight
Pub 2	Curved	Curved	Straight	Straight
Pub 3	Straight	Straight	Curved	Normal glass range^a^

Curved and straight refer to the shape of experimenter-supplied glassware

^a^Indicates where the public house used its normal range of glassware

Ethics approval for the study was obtained from the Faculty of Science Ethics Committee at the University of Bristol (reference number: 2502146682). The study was conducted in accordance with the Declaration of Helsinki (2013) principles.

### Study sites

Three public houses owned by Dawkins Ales located in Bristol, UK, took part in the study over the course of two weekends in April 2014. The three public houses were run by individual landlords, relatively small, with a capacity of 25–75 people.

### Materials

**Fig. 1:**
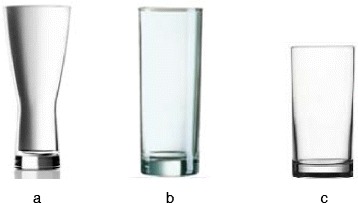
Shapes of glasses. **a** Sahm’s Tokyo glass, pint glass: Art. Nr. 1005428 and half-pint glass: Art. Nr. 1005930. **b** Arcoroc’s Geo glass 20 oz (58.5 cl) glass. **c** Paşabahçe’s highball long drinking glass can hold 285 cc, 9.5 oz (US) and 10.25 oz (UK)

Straight-sided and curved pint and half-pint glasses were delivered to the public houses by the experimenters. The curved pint and half-pint glasses were Tokyo style glasses (Fig. 1a, [36]) designed and supplied by Sahm, whereas the straight pint and half-pint glasses were ‘highball’ style glasses, designed and supplied by Arcoroc Professional (Fig. 1b, [37]) and Pasabahce (Fig. 1c, [38]), respectively. Alcoholic beverages were supplied by the public houses as part of their usual trade.

### Procedure

The owner of Dawkins Ales was identified as running several local public houses, and the purpose of the study was explained to him. He agreed to introduce the study team to public houses that formed part of the Dawkins Ales group and encourage them to support the study. Through this introduction, the study team explained the study to individual landlords, who made the final decision whether or not to participate. All landlords approached agreed for their public house to participate in the study. They were also informed of an unrelated experiment investigating the effects of drinking alcohol on ratings of attractiveness that the experimenters wanted to carry out in their public houses a month after the feasibility study, and all landlords agreed to this request.

Glasses were delivered to each of the three public houses by the experimenters during the week before the first weekend and were changed midweek. Public house landlords were requested to serve all beer/cider from the supplied glasses unless patrons explicitly requested another glass. At the end of each weekend, total monetary takings (excluding takings for food) for each of the three public houses were obtained from the landlords, from a till printout. At the end of the final weekend, glasses were removed from the public houses and original glasses were restocked.

Informal feedback from the brewer and landlords was obtained by two experimenters after completion of the feasibility study but before the attractiveness experiment. It was a face-to-face discussion, rather than a formal qualitative interview, and was not audio recorded, although notes were taken. All landlords were asked specific questions regarding the study logistics and how these could have been improved, the suitability of the incentive structure, what would encourage them to participate in future studies and the experience of customers during the trial. After these topics were covered, there was a free-form discussion. The feedback supplemented what was learned from the rest of the trial and provided the landlord’s perspective.

### Statistical analysis

Monetary takings data from three public houses were recorded for straight glass weekends and curved glass weekends. The difference in average takings across the straight and curved glass weekends was assessed using a paired-samples *t* test. Due to the commercially sensitive nature of reporting absolute monetary takings, the mean difference and 95 % confidence intervals (CI) were calculated based on difference scores between takings from straight glass weekends and curved glass weekends, which were then converted into percentage change.

To investigate if monetary takings are an accurate proxy for alcohol consumption, data were obtained from a public house not involved in the feasibility study over a 2-week period in January 2015. Monetary takings for three beverage categories (beer/cider, wine and spirits) were extracted from itemised till printouts. Units of alcohol per beverage were calculated by obtaining alcohol by volume (ABV) percentages and volume amounts from the public houses’ drinks list. Separate Pearson product-moment correlations were calculated to assess the relationship between units of alcohol sold and monetary takings for beer/cider (*N* [number of drinks] = 19), spirits (*N* = 24) and wine (*N* = 30).

## Results

This section is structured following recommended guidelines for reporting feasibility studies [39].

### Acceptability

Communication with one landlord broke down during the study, and he was not willing to participate on weekend 2, although he still supplied monetary takings data for that weekend. The storage space for glassware was very limited in this public house, and the normal glassware range had to be stored off-site for weekend 1 of the study. This resulted in a laborious process of boxing all of his glassware. Although assistance was provided by the study team, this was felt to be a source of disruption by the landlord. Also, some of his regular clientele expressed dissatisfaction with the new glassware.

This feasibility study was run as part of a two-study collaboration with the public houses. The other study was an unrelated experiment investigating the effects of alcohol consumption on ratings of attractiveness. This experiment was run in the same establishments but was conducted 1 month later. Critically, unlike the attractiveness study, the feasibility study was not publicised, so as not to distort the behaviour of customers at the participating public houses. The quid pro quo of granting the public houses’ publicity through local and university media to both obtain a healthy number of participants for the other study and temporarily increase their custom proved effective in obtaining and maintaining their support for the feasibility study.

### Implementation

The logistics of delivering and collecting glassware were negotiated with each landlord. One public house required experimenters to store their glassware off-site during the study. The dishwasher in one public house was too small to wash the experimenter’s curved full-pint glasses, and the public house could not use them (see Table 1), so it used its standard glass range instead on weekend 1.

### Demand

Based on feedback from landlords, a small number of patrons were unhappy with the new glassware and requested their normal glass. These requests were honoured. However, the majority of customers during the trial accepted the new glassware.

### Practicality

Monetary takings were a practical way of measuring alcohol consumption without disruption to normal trade. The landlords were forthcoming with the monetary takings shortly after the weekends when the study took place. The information supplied by the public houses included monetary amounts for all sales (excluding food) over the study weekends. There were varied opening times for each of the three public houses; however, common, overlapping opening times of 5 to 11 p.m. were used when obtaining data on monetary takings. The experimenters’ curved glasses were not used in two public houses (see Table 1). Critically, the intervention (straight) glasses were used by all three public houses. The comparison between straight glasses and the landlord’s standard glass range was deemed valid (as our comparison is with usual practice) for the purposes of the feasibility study. The difference in monetary takings was estimated using a paired-samples *t* test, which indicated that takings were reduced by 24 % (95 % CI 77 % reduction to 29 % increase) for the weekends when alcohol was served in straight glasses compared to the weekends when alcohol was served in curved glasses.

Pearson product-moment correlations were used to investigate if monetary takings are an accurate proxy for alcohol consumption. Results provided strong statistical evidence for a positive correlation between units of alcohol sold and monetary takings for each beverage category (beer/cider: *r* [17] = 0.996, *p* ≤ 0.001; spirits: *r* [22] = 0.986, *p* ≤ 0.001; wine: *r* [28] = 0.986, *p* ≤ 0.001).

### Integration

The introduction of new glassware was integrated into two public houses’ normal trade successfully with appropriate planning and communication. As described above, one landlord felt that exchanging glassware was disruptive to his normal business. A convenient time for delivery and collection of glassware had to be negotiated with each public house. The glassware supplied by experimenters did not contain any nucleation, and it was noted by landlords during post-study feedback that glasses need to be nucleated for lager and cider beverages to better integrate into the public houses’ normal trade.

### Expansion

A sample size calculation for a future full trial was carried out based on the results of the paired-samples *t* test. A sample size of 30 public houses and bars would be sufficient to detect a moderate to strong difference in monetary takings of at least 0.6 standard deviations with 90 % power at the 5 % significance level (assuming a correlation of *r* = 0.65 in takings between the two periods) [40].

## Discussion

We have demonstrated that, with detailed planning and good communication with public house staff, naturalistic studies of this nature can be run effectively. Communication with individual proprietors of public houses was essential to keep them engaged with the study. However, some important lessons were also learned. Communication with one landlord broke down during the study, and he was not willing to participate further. The dishwasher in one public house was too small to wash the experimenter’s curved full-pint glasses, and the public house could not use them (see Table 1), so it used its standard glass range instead. The majority of customers during the trial accepted the newly shaped glassware, although some did express dissatisfaction. The collection of monetary takings from each landlord was straightforward and was carried out by an experimenter after the first weekend and at the end of the study. Overall, the brewer and landlords were satisfied with how the study was carried out and there is potential for further collaboration.

The breakdown of communication with one landlord resulted in him not participating on weekend 2 of the study. Experimenters were relying on the brewer to maintain good communication with his landlord regarding the purpose and importance of the study. Good communication is needed between the study team and each individual landlord directly to foster loyalty to any future study. Experimenters need to engage each landlord with the study and explain incentives to them effectively, so that potential benefits to landlords are clear. Being a small public house with regular clientele, some of them expressing dissatisfaction with the new glassware, may have been an important factor in his decision not to participate on weekend 2.

The consensus from our post-study feedback with the brewer and landlords was that no reimbursement for involvement was necessary except for being supplied with new glasses at the end of the study. However, in our opinion, the brewer and landlords would have been less keen to participate if there was not a second event that provided publicity and increased custom for their public houses. If this type of incentive is not feasible for a particular study, it may need to be replaced to encourage participation from public houses in the future. This could include new glassware provided free of charge, and/or the public houses’ participation could be publicised via blogs and press releases after data collection has concluded. The optimal method of compensation should be discussed with landlords. If any intervention serves to reduce monetary takings, it would seem ethical and appropriate to offer some financial reimbursement for this, given the competitive nature of the industry (particularly when participating public houses are part of a relatively small local brewer, as in this case). Hopefully, if a commitment to offer financial reimbursement for loss of earnings was made before a future trial, this may alleviate the concern of landlords of maintaining equivalency of earnings.

A public house’s dishwasher and shelving units should be inspected to ensure they are fit for purpose before participation in any future study. Storing one public house’s original stock of glasses off-site during the study was not an issue; however, if more public houses are involved in future studies, this may be difficult. Where possible, public houses should be able to store all glassware on-site. If this is not possible, contingency plans should be put in place. The storage space of each public house should be assessed at an early stage in future studies. If glasses are going to be given to public houses as an incentive to participate in future studies, this may be less of an issue. Assistance was provided by one experimenter to help stock and wash the glasses which was appreciated by landlords. Further assistance from the study team may be needed in future studies so that transfers of glassware are less disruptive.

There was a reduction in takings on weekends when straight glasses were stocked in the three public houses compared to when curved glasses were stocked. Although the mean difference (24 % reduction) was imprecise with wide confidence intervals, it is worth noting that it was in the same direction as a previous laboratory study investigating the effect of glass shape on the drinking rate of an alcoholic beverage [20]. The wide confidence interval (95 % CI 77 % reduction to 29 % increase) suggests a large variability between the three public houses, and these results should be considered with caution, as the study was not designed to support hypothesis testing. The main purpose in collecting the results was to inform the design of a future full trial. Dawkins Ales, which owns all three public houses, requested that monetary takings not be made public, due to the commercial sensitivity of this information; therefore, reporting aggregated results was not possible. However, if there was a larger number of participating public houses involved, as would be the case in future studies, reporting aggregate amounts of monetary takings would be possible with appropriate approval because it would be harder to infer the takings of individual public houses in this situation.

Results from the post-study investigation into the accuracy of monetary takings as a proxy for alcohol consumption showed consistently strong, positive correlations between units of alcohol sold and monetary takings for different beverage categories. This suggests that monetary takings are an accurate proxy for alcohol consumption based on these data. A limitation of the data used was the inability to calculate the amount of alcohol sold on a daily basis within the 2-week window; therefore, a correlation between units of alcohol consumed and total monetary takings could not be calculated. A standard measure of alcohol units can be compared across sites to evaluate alcohol use in different conditions (straight vs curved) provided that data on units of alcohol in beverages of interest are available to researchers. In future studies, landlords should be requested to send their takings (preferably broken down by beverage type) on a weekly/periodic basis to experimenters via email or a collection of them could also be arranged. Many modern tills have the capability to break down purchases into different drink types, and this should be utilised in future studies. This would allow sales of soft drinks to be accurately separated from alcoholic drinks and changes in the sales of soft drinks to be monitored over the duration of a future trial. Another option is to take an inventory of alcohol (e.g. number of kegs, bottles etc.) although public houses may be reluctant to give such detailed information. If this is the case, monetary takings can be used effectively as a proxy for alcohol consumption. A balance must be kept between accuracy of alcohol consumption and maintaining a naturalistic drinking environment.

Customers taking their custom elsewhere is a potential alternative explanation for the reduction in alcohol consumption during the study when public houses were stocked with straight glasses. This would appear to be a reduction in consumption in a trial, but would not in fact reflect a reduction in individual-level consumption. This would be difficult to monitor in a real-world environment. However, the low percentage of patrons objecting to the straight glasses in this trial suggests that the level of customer dissatisfaction may not deter public houses from participating especially with the agreement to compensate for any lost profits during a trial.

Studies of this nature can be run on a relatively low research budget. Data collection costs can be kept to a minimum at each study site, since the intervention can be delivered within public houses and bars as part of their routine trade. Post-study feedback suggested that two public houses found the intervention practical to implement. The public house who withdrew from the study after weekend 1 found aspects of the study impractical to implement, namely disruption caused by changing glassware and customer dissatisfaction with experimenter-supplied glassware. Sensitivity to these types of issues needs to be paramount when assessing the practicality of future study designs.

Glassware supplied by the study team did not include any branding, nucleation or volume labelling, which are all common features on glassware used in on-trade premises. As the intervention (unbranded) glassware was not used on two of the weekends, it is possible that these aesthetic factors may have influenced alcohol consumption, rather than the structural properties of the glass. This is a point to consider when trying to integrate into the normal trade of a public house, since they may be reluctant to stock glasses without these design features for a longer period, in which case some elements (e.g. nucleation and volume labelling) may have to be applied to all glasses. It should be considered that some consumers who expressed dissatisfaction with experimenter-supplied glassware in this study may have done so due to the lack of these common features of modern glassware being present. Considerations around glass design (e.g. branding, nucleation) should be responsive to these views.

If the intervention is shown to be effective by reducing alcohol consumption, it would need to be implemented legislatively, due to the demand to implement the intervention voluntarily by public houses predicted to be low. In the UK, the 2003 Licensing Act [41] afforded powers to local licensing authorities to issue alcohol licences and enforce the conditions of the licence in their area. This change has made licensing more local and flexible to the needs of the local community. It has also made the process more responsive to emerging evidence. Alcohol licensing conditions are not subject to the same regulatory framework as, for example, treatments within the National Health Service, meaning that evidence of efficacy can be directly translated into policy much more rapidly. It is conceivable that the evidence from a future study could be implemented in local authority licensing policies within 2–5 years of the end of the study (depending on where in the licensing cycle the evidence becomes available). If results show that straight glasses reduce consumption, a local licensing authority would be able to add a requirement to stock straight glasses to its ‘menu’ of licensing conditions which it can require premises to accept in order to be granted a licence. When an existing premise applies to vary its licence, a responsible authority can demand certain conditions to be met in order for the variation to be granted. Any person or responsible authority (e.g. the local police force) can also apply to the licensing authority for a review of an existing licence, with the aim of amending its conditions. If evidence shows that straight glasses reduce consumption, the police or local licensing authority may deem it worthwhile to require that more straight glasses be stocked in existing licensed premises to bring about a reduction in crime and public disorder associated with alcohol misuse [42–44]. Critically, the intervention is one which, if mandated would not impose additional direct costs on public houses and bars, since the glassware that constitutes the intervention is no more expensive than the existing glassware. Moreover, since glassware is replaced regularly (due to breakages etc.), any transition would have minimal impact.

A limitation of the study was that data on the usual business of the public houses were not collected. However, we have no particular reason to think the weekends were not representative of normal business when the study took place. Another limitation of the study was that we explicitly targeted on-trade consumption of alcohol, but individuals are increasingly consuming alcohol at home [1]. However, if consumption can be lowered in the on-trade market, this would still have a significant impact on public health. Also, the hypothesised impact of straight glasses is not exclusive to on-licence premises, and there is potential for a similar effect in slowing drinking rate in the home.

Further studies should expand in scope to include other public houses over longer periods of time to get a more comprehensive picture of the effect of glass shape on alcohol intake. We suggest that the indirect measurement of alcohol consumption, using monetary takings from itemised till receipts for alcoholic beverages, may be an appropriate outcome measure in future studies. On the basis of our experience in this feasibility study and the sample size calculation for a future trial, we estimate that a 6-month data collection period in 30 public houses and bars would be sufficient to detect a difference in monetary takings. Collaborating with larger chains of public houses in the future would present unique challenges. The increased number of staff working in these establishments would involve putting more trust in management to communicate effectively with their employees. More glassware would be required, and a more substantial logistical effort would be needed to transport and stock these glasses. Extra personnel would be needed to carry this out. It may be more difficult to get larger chains of public houses involved in public health research on their premises, given that stocking straight glasses would impact on their business in the long term. It may be more fruitful to engage with public houses with a community ethos rather than a high-volume business model. Other key stakeholders, including local authorities and relevant trade associations, have also pledged their support for future studies, and this should aid recruitment efforts. Our research group is consistently forging links with industry partners which will pay dividends when recruiting for future studies. To avoid attrition in future studies, open communication should be maintained with each individual landlord so that any issues and concerns can be dealt with as soon as they arise. A periodic meeting between staff of public houses and experimenters during future studies is advisable. Nevertheless, study designs should incorporate the possibility of attrition due to participating public houses dropping out over the course of the study. Advantages such as publicity and new glassware at low or no cost to their public house should be emphasised to foster loyalty to future studies.

## Conclusions

It is feasible to manipulate the type of glasses in a public house provided there is detailed planning and clear communication with landlords. It is feasible to monitor alcohol intake of customers via monetary takings with no disruption to normal trade. Brewery owners and public house landlords will participate and allow studies in their establishments given the appropriate incentive structure. The logistical challenges encountered during this trial and our proposed solutions will inform other study teams aiming to carry out naturalistic studies in public houses. It is pivotal to establish what types of study designs can be executed and what interventions can be tested in public houses. The efficacy of potential interventions need to be evaluated in ‘real-world’ environments in order to persuade local licensing bodies to implement emerging evidence into local licensing policy. Choice architecture interventions—such as modifying glass shape—can contribute to population-level reductions in excessive alcohol consumption.

## References

[1] British Medical Association (2008). Alcohol misuse: tackling the UK epidemic.

[2] Lifestyle Statistics, Health & Social Care Information Centre (2014). Statistics on alcohol England.

[3] Williams R, Aspinall R, Bellis M, Camps-Walsh G, Cramp M, Dhawan A (2014). Addressing liver disease in the UK: a blueprint for attaining excellence in health care and reducing premature mortality from lifestyle issues of excess consumption of alcohol, obesity, and viral hepatitis. Lancet.

[4] Huang C (2003). Econometric models of alcohol demand in the United Kingdom.

[5] Patra J, Giesbrecht N, Rehm J, Bekmuradov D (2012). Are alcohol prices and taxes an evidence-based approach to reducing alcohol-related harm and promoting public health and safety? A literature review. Contemp Drug Probl.

[6] Holmes J, Meng Y, Meier PS, Brennan A, Angus C, Campbell-Burton A (2014). Effects of minimum unit pricing for alcohol on different income and socioeconomic groups: a modelling study. Lancet.

[7] Room R, Babor T, Rehm J (2005). Alcohol and public health. Lancet.

[8] Department of Health (2010). Healthy lives, healthy people: our strategy for public health in England.

[9] Cabinet Office Behavioural Insights Team (2010). Applying behavioural insight to health.

[10] Hollands GJ, Shemilt I, Marteau TM, Jebb SA, Kelly MP, Nakamura R (2013). Altering micro-environments to change population health behaviour: towards an evidence base for choice architecture interventions. BMC Public Health.

[11] Hofmann W, Friese M, Wiers RW (2008). Impulsive versus reflective influences on health behavior: a theoretical framework and empirical review. Health Psychol Rev.

[12] Marteau TM, Ogilvie D, Roland M, Suhrcke M, Kelly MP (2011). Judging nudging: can nudging improve population health?. BMJ.

[13] Marteau TM, Hollands GJ, Fletcher PC (2012). Changing human behavior to prevent disease: the importance of targeting automatic processes. Science.

[14] Gueguen N, Le Guellec H, Jacob C (2004). Sound level of background music and alcohol consumption: an empirical evaluation. Percept Mot Skills.

[15] Gueguen N, Jacob C, Le Guellec H, Morineau T, Lourel M (2008). Sound level of environmental music and drinking behavior: a field experiment with beer drinkers. Alcohol Clin Exp Res.

[16] Hughes K, Quigg Z, Eckley L, Bellis M, Jones L, Calafat A (2011). Environmental factors in drinking venues and alcohol-related harm: the evidence base for European intervention. Addiction.

[17] Wansink B, van Ittersum K (2005). Shape of glass and amount of alcohol poured: comparative study of effect of practice and concentration. BMJ.

[18] Jones SC, Gregory P (2009). The impact of more visible standard drink labelling on youth alcohol consumption: helping young people drink (ir)responsibly?. Drug Alcohol Rev.

[19] Stockwell T (2006). A review of research into the impacts of alcohol warning labels on attitudes and behaviour.

[20] Attwood AS, Scott-Samuel NE, Stothart G, Munafò MR (2012). Glass shape influences consumption rate for alcoholic beverages. PLoS One.

[21] Quigley BM, Collins RL (1999). The modeling of alcohol consumption: a meta-analytic review. J Stud Alcohol.

[22] Lau-Barraco C, Braitman AL, Leonard KE, Padilla M (2012). Drinking buddies and their prospective influence on alcohol outcomes: alcohol expectancies as a mediator. Psychol Addict Behav.

[23] Christiansen BA, Goldman MS, Inn A (1982). Development of alcohol-related expectancies in adolescents: separating pharmacological from social-learning influences. J Consult Clin Psychol.

[24] Brown SA, Christiansen BA, Goldman MS (1987). The alcohol expectancy questionnaire: an instrument for the assessment of adolescent and adult alcohol expectancies. J Stud Alcohol.

[25] Fromme K, Stroot EA, Kaplan D (1993). Comprehensive effects of alcohol: development and psychometric assessment of a new expectancy questionnaire. Psychol Assess.

[26] Fromme K, D'Amico EJ (2000). Measuring adolescent alcohol outcome expectancies. Psychol Addict Behav.

[27] Patrick ME, Wray-Lake L, Finlay AK, Maggs JL (2010). The long arm of expectancies: adolescent alcohol expectancies predict adult alcohol use. Alcohol Alcohol.

[28] Natvigaas H, Leigh BC, Anderssen N, Jakobsen R (1998). Two-year longitudinal study expectancies and drinking adolescents of alcohol among Norwegian adolescents. Addiction.

[29] Reis J, Riley WL (2000). Predictors of college students’ alcohol consumption: implications for student education. J Genet Psychol.

[30] Martin CM, Hoffman MA (1993). Alcohol expectancies, living environment, peer influence, and gender: a model of college-student drinking. J Coll Stud Dev.

[31] Cooper ML, Russell M, Skinner JB, Windle M (1992). Development and validation of a three-dimensional measure of drinking motives. Psychol Assess.

[32] Jones BT, Corbin W, Fromme K (2001). A review of expectancy theory and alcohol consumption. Addiction.

[33] Southwick LL, Steele CM, Marlatt GA, Lindell MK (1981). Alcohol-related expectancies: defined by phase of intoxication and drinking experience. J Consult Clin Psychol.

[34] Greenfield TK, Harford TC, Tam TW (2009). Modeling cognitive influences on drinking and alcohol problems. J Stud Alcohol Drugs.

[35] Pabst A, Baumeister SE, Kraus L (2010). Alcohol-expectancy dimensions and alcohol consumption at different ages in the general population. J Stud Alcohol Drugs.

[36] Sahm’s Tokyo style glass. http://www.sahm.de/en/categories/beer/products/tokyo-v2?type=tumbler. Accessed 1 July 2015.

[37] Arcoroc professional’s geo glass. http://ecatalogue.arcoroc.com/viewer/AN050913_@CatArcBis_pdf_302/. Accessed 1 July 2015.

[38] Pasabahce’s highball glass. http://www.pasabahce.com/urunler/kataloglar/service-line/hiball/41412.aspx. Accessed 1 July 2015.

[39] Bowen DJ, Kreuter M, Spring B, Cofta-Woerpel L, Linnan L, Weiner D (2009). How we design feasibility studies. Am J Prev Med.

[40] Machin D, Campbell MJ, Fayers PM, Pinol A (1997). Sample size tables for clinical studies.

[41] Act of UK Parliament. Licensing Act 2003. http://www.legislation.gov.uk/ukpga/2003/17/contents Accessed 17 July 2015.

[42] Collins JJ (1981). Drinking and crime: perspectives on the relationship between alcohol consumption and criminal behaviour.

[43] HM Government (2007). Safe, sensible, social: the next steps in the national alcohol strategy.

[44] Plant M, Plant M, Thornton C (2002). People and places: some factors in the alcohol-violence link. J Subst Use.

